# CO_3+1_ network formation in ultra-high pressure carbonate liquids

**DOI:** 10.1038/s41598-019-51306-6

**Published:** 2019-10-28

**Authors:** Martin Wilding, Paul A. Bingham, Mark Wilson, Yoshio Kono, James W. E. Drewitt, Richard A. Brooker, John B. Parise

**Affiliations:** 1 Materials and Engineering Research Institute, Sheffield Hallam University, Howard Street, Sheffield, S1 1WB UK; 2 Department of Chemistry, Physical and Theoretical Chemistry Laboratory, University of Oxford, South Parks Road, Oxford, OX1 3QZ UK; 3 Geophysical Laboratory, Carnegie Institute of Washington, 9700 S. Cass Avenue, Argonne, IL 60439 USA; 4 School of Earth Sciences, University of Bristol, Queens Road, Wills Memorial Building, Bristol, BS8 1RJ UK; 5 SUNY, Stony Brook, NY 11794 USA; 6 Present Address: University of Manchester at Harwell, Diamond Light Source, Didcot, Oxfordshire OX11 0DE UK; 7 Present Address: Geodynamics Research Center, Ehime University, 2-5 Bunkyo-cho, Matsuyama, Ehime 790-8577 Japan

**Keywords:** Petrology, Computational chemistry, Structure of solids and liquids

## Abstract

Carbonate liquids are an important class of molten salts, not just for industrial applications, but also in geological processes. Carbonates are generally expected to be simple liquids, in terms of ionic interactions between the molecular carbonate anions and metal cations, and therefore relatively structureless compared to more “polymerized” silicate melts. But there is increasing evidence from phase relations, metal solubility, glass spectroscopy and simulations to suggest the emergence of carbonate “networks” at length scales longer than the component molecular anions. The stability of these emergent structures are known to be sensitive to temperature, but are also predicted to be favoured by pressure. This is important as a recent study suggests that subducted surface carbonate may melt near the Earth’s transition zone (~44 km), representing a barrier to the deep carbon cycle depending on the buoyancy and viscosity of these liquids. In this study we demonstrate a major advance in our understanding of carbonate liquids by combining simulations and high pressure measurements on a carbonate glass, (K_2_CO_3_-MgCO_3_) to pressures in excess of 40 GPa, far higher than any previous *in situ* study. We show the clear formation of extended low-dimensional carbonate networks of close CO_3_^2−^ pairs and the emergence of a “three plus one” local coordination environment, producing an unexpected increase in viscosity with pressure. Although carbonate melts may still be buoyant in the lower mantle, an increased viscosity by at least three orders of magnitude will restrict the upward mobility, possibly resulting in entrainment by the down-going slab.

## Introduction

Changes in glass and liquid structure in response to the application of high pressures have engaged a wide variety of disciplines including condensed matter physics, materials science and geosciences. These changes can be used to develop an understanding of the physical behaviour of liquids at high pressure and the associated chemical and geochemical processes.

One particularly important class of liquids are carbonates. These are significant geochemical agents of transport in the deep Earth (particularly between the upper and lower mantles)^1–5^. They are chemically stable to pressures of at least 50 GPa^1,4^ and electrical conductivity studies indicate the existence of a carbonate reservoir in the lower mantle^6–9^. Despite this critical role, the behaviour of carbonate liquids at high pressure and temperature is not well-understood and remains relatively unexplored. There are few measurements of carbonate liquid structure at high pressure, and although there have been recent *in situ* studies of the CaCO_3_ liquid structure^10^ as well as density and viscosity measurements of other carbonate liquids^11,12^, these measurements are limited to pressures of less that 10 GPa since the liquids are hard to encapsulate. Studies of liquids at ambient pressure (including nitrates and carbonates^13–15^) have shown the formation of low-dimensional structures and networks characterized by a second length scale, and would suggest the structural response at higher pressures may be more substantial than these *in situ* studies would indicate. High pressure amorphous forms of nitrogen and CO_2_ have been reported^16–20^ which also indicate formation of polymerised networks at very high pressures(≥50 GPa).

In this study we will explore the high pressure structure of carbonate liquids by using *in situ* X-ray diffraction measurements of a rare carbonate glass combined with advanced molecular dynamics simulations performed on the equivalent liquid. Glasses are often used as proxies for liquids since access to high temperature liquid structures by either diffraction or spectroscopic techniques is challenging. This is particularly the case for high pressure studies since the contribution from sample environment can dominate these measurements. Although *in situ* high pressure and high temperature liquid structure studies remain few and far between^10,21–24^, studies of glass structure can be more readily undertaken and there have been a number of recent high-pressure glass studies at ambient temperature, including SiO_2_, GeO_2_ and B_2_O_3_^25–27^. These studies tend to show limited changes in structure at pressures less than 20 GPa whilst more dramatic changes in the structure of GeO_2_, for example, have been demonstrated at *ultra high* pressures (≥70 GPa)^28^. These latter measurements use specially developed high pressure techniques^28^ and in this study the same ultra-high pressure technology is used to determine the high pressure structure of a carbonate glass to pressures of 44 GPa.

Carbonate glasses are very rare. The formation of a glass in the system K_2_CO_3_-MgCO_3_ was, however, reported by Eitel and Skaliks as long ago as 1929^29^ as a passing observation and has, with a few exceptions^30,31^ received little attention. Glass in this system can be formed in a deep eutectic region at pressures of ~50 MPa^32,33^. The elevated pressure is believed to prevent the carbonate decomposing. Carbonates, along with other ionic glass formers such as sulphates^34,35^ and nitrates^36^, lack conventional network-formers such as silicate tetrahedra and there is considerable speculation about how these exotic glasses form, and their structure. In theory, the ionic nature of the carbonate anion should be dictated by the electronic structure in which all the “bonding” oxygen orbitals are incorporated into CO p*π* and s*σ* bonds leaving none for covalent interactions. As such, they should not form the covalently-bonded polymerized network normally required for a melt structure to quench to a glass^1^.

Spectroscopic studies of the K_2_CO_3_-MgCO_3_ glass^30,31^ indicate the presence of two structurally distinct populations of carbonate anions. Genge *et al*.^31,37^ suggest that the more symmetrical units form a flexible network that comprises carbonate anions with bridging, strongly interacting metal cations (here Mg^2+^) while non-bridging species (here K^+^) modify the network and are associated with distorted carbonate groups. It has been suggested that^34–36^ glass formation in sulphate and nitrate systems requires the presence of two different cations with different field strengths and different degrees of polarizibility. This proposed structure of K_2_CO_3_-MgCO_3_ glass is significantly more complicated than simple ionic molten salt models would predict and is defined by the flexibility of the molecular anion. The non-ionic behavior of carbonate anions has also been noted when dissolved in highly polymerized aluminosilicate liquids. Vibrational spectra and simulations suggest that carbonate anions form bridging units within the fully polymerized aluminosilicate network^38–41^ and in more de-polymerized melts they may form a distorted sub-lattice network as a precursor to phase separation of an immiscible carbonate liquid^42,43^. Molecular dynamics (MD) simulations, used to explore the high pressure behavior of carbonate liquids^37^, also indicate that the flexibility of the carbonate anion strongly influences the structure and dynamics of carbonate liquids and their glass formation.

The structure and structure-related properties of carbonate and nitrate liquids have been the focus of recent studies wherein advanced ultra-high pressure synchrotron techniques were combined with state-of-the-art molecular dynamics simulation using potentials that permit the molecular anions to be inherently flexible^13–15^. Previously we have shown that molten carbonates are characterized by the emergence of low-dimensional structures that are temperature-dependent and have a size and extent that can be directly correlated with the liquid dynamics and liquid *fragility*^15^. In the present study we explore the high pressure behavior of carbonate liquids using a similar approach. The X-ray measurements were performed on the K_2_CO_3_-MgCO_3_ glass over a large pressure range with concomitant MD simulations undertaken at different liquid densities to establish the pressure-dependent structural trends.

## Results

**Figure 1:**
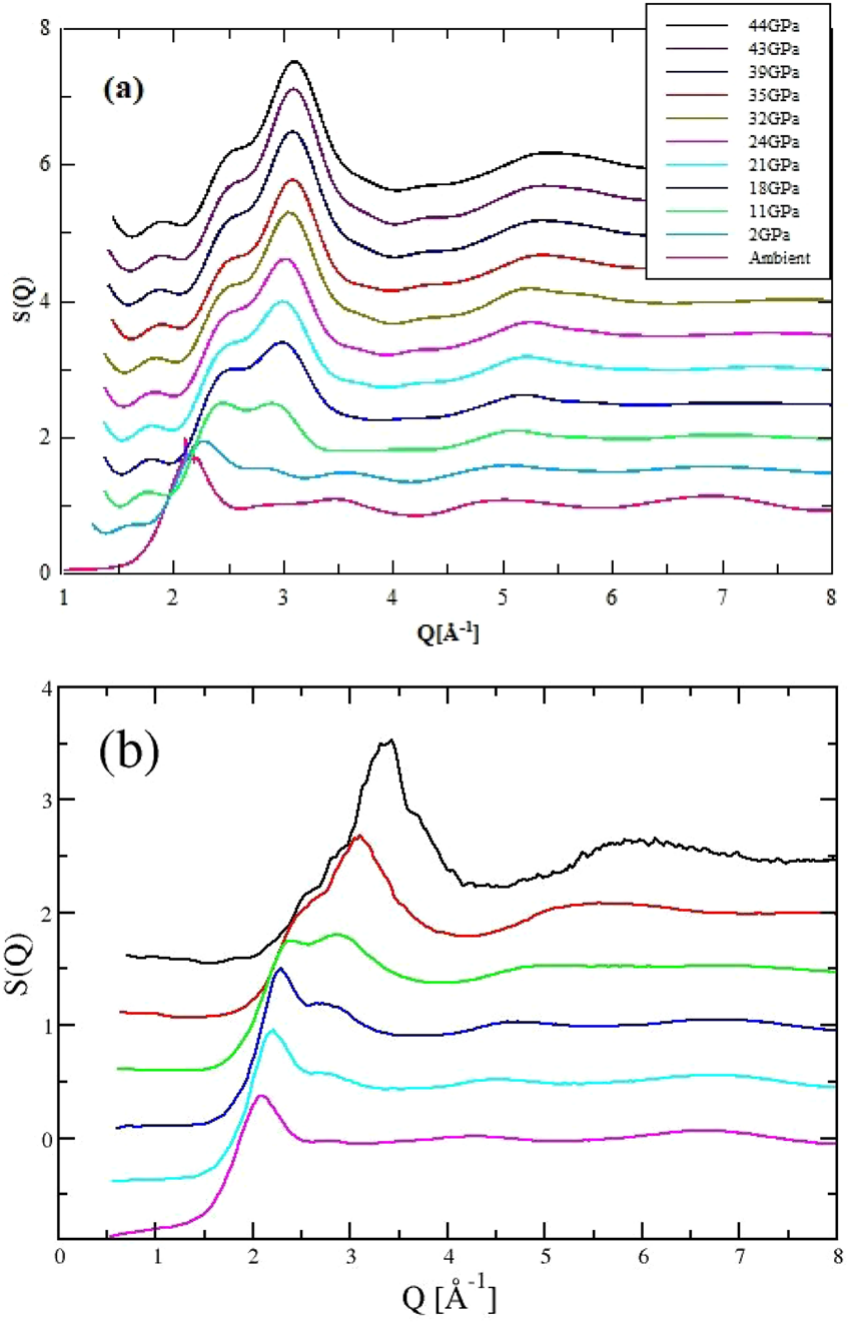
(**a**) Experimentally-determined X-ray total structure factors, *S*(*Q*), taken at the pressures indicated and presented as a error-weighted smoothed spline fit to individual detector segments. (**b**) Total X-ray structure factors obtained from molecular dynamics computer simulation at six densities (increasing from bottom to top, (in molecules per Å^3^, *n*_0_ = 0.00572, 0.00656, 0.00722, 0.00797, 0.00979 and 0.0122) In both panels successive curves are offset along the abscissa for clarity.

High pressure studies of carbonate glass were performed with energy dispersive diffraction at beamline 16BMB (HPCAT) at the Advanced Photon Source (APS). The configuration at this beamline enables the specially developed double-stage large volume (Paris-Edinburgh type) press to be integrated^11,28,44–46^. The total X-ray structure factor, *S*(*Q*), for each pressure point was obtained using the multi-angle energy dispersive technique^28,47^ (see SI Fig. S1) with spline-smoothed curves produced by correcting the data from each detector bank and normalising to the white X-ray beam^28,47^. While there are clear overlaps between the detector sections (see SI Fig. S2), these do not correlate with the underlying changes in glass structure. In Fig. 1 *S*(*Q*) for each pressure is presented as an inverse Fourier-filtered function, obtained by back-transform of the real space data (see SI). The first peak in the diffraction pattern is the prominent peak at *Q* ~2.1 Å^−1^ and will be a superposition of the dominant partial contributions. This is indicative of intermediate range order in the glass whilst the oscillations at higher *Q* correspond to short range contributions. Faber-Ziman X-ray weightings indicate that the total scattering pattern will be dominated by the partial contributions from K-O, O-O and K-K pairs. The contributions from atom pairs containing C or Mg atoms are weak. The diffraction patterns in Fig. 1 are, therefore, expected to be dominated by the changes in K-O sub-density.

As pressure is increased (Fig. 1) the immediate response of the glass structure is a shift to higher Q of the first peak in the diffraction pattern. This corresponds to a shift to lower *r* in real space and is consistent with an increase in the glass density. There are additional and more substantial structural changes as pressure is increased. The most obvious is the development of a shoulder to the first peak at *Q* ~2.9 Å^−1^ which first appears at 0 < *p* < 2 GPa and becomes increasingly dominant, shifting to higher *Q*, as the pressure increases. The partial contribution of the C-O pairs to *S*(*Q*) is low and it is difficult to draw definitive interpretations about the their contribution without the aid of a structural model. The pair distribution functions (shown as *D*(*r*) = *r*(*G*(*r*) − 1)) for the carbonate glass at different pressures (see SI Fig. S2) do, however, show a peak at *r* ~ 1.3 Å that corresponds to the C-O peak for carbon coordinated by three oxygen atoms. As pressure is applied, this peak decreases in intensity and becomes broader, and is barely resolved at the higher pressure suggesting changes in the short range order of the carbonate anion. There are also overlapping, partly resolved peaks at higher *r* which merge to form a single peak at *r* ~ 3 Å at pressures greater than 11 GPa (see SI). In Fig. 1 it is clear that the changes in *S*(*Q*) are continuous with pressure, with no evidence for a discontinuous or *polyamorphic* transition, although, as with CaCO_3_, different amorphous structures are progressively stabilised^48^. It is also important to note that the changes in structure reflect changes in both the intermediate- and short-range length-scales, and indicate an increase in ordering with pressure evidenced by the higher *r* contributions. Molecular dynamics simulations of the equivalent liquid in this system facilitate a more effective interpretation of the response to pressure.

In previous work molecular dynamics simulations have been performed on carbonate liquids using a potential developed by Tissen and Janssen, of the Born-Huggins-Mayer form^49^. The trigonal geometry of the carbonate anion is imposed by employing harmonic springs that act between C-O and O-O pairs^13^. In later studies^13,15^ we developed an approach that allows both flexibility of the molecular anion and fluctuation of the internal charge distribution^50–56^. In the simulations carried out here we fix the charge distribution on the anion (see SI). The simulations have been carried out at a fixed temperature (of *T* = 1800 K) and constant volume, with the simulations performed at different densities to evaluate the influence of pressure on the liquid carbonate structure. The goal of the simulations is not to reproduce each state point precisely but to identify the trends in the carbonate liquid structure that are seen in the equivalent glass structures as pressure is increased.

**Figure 2:**
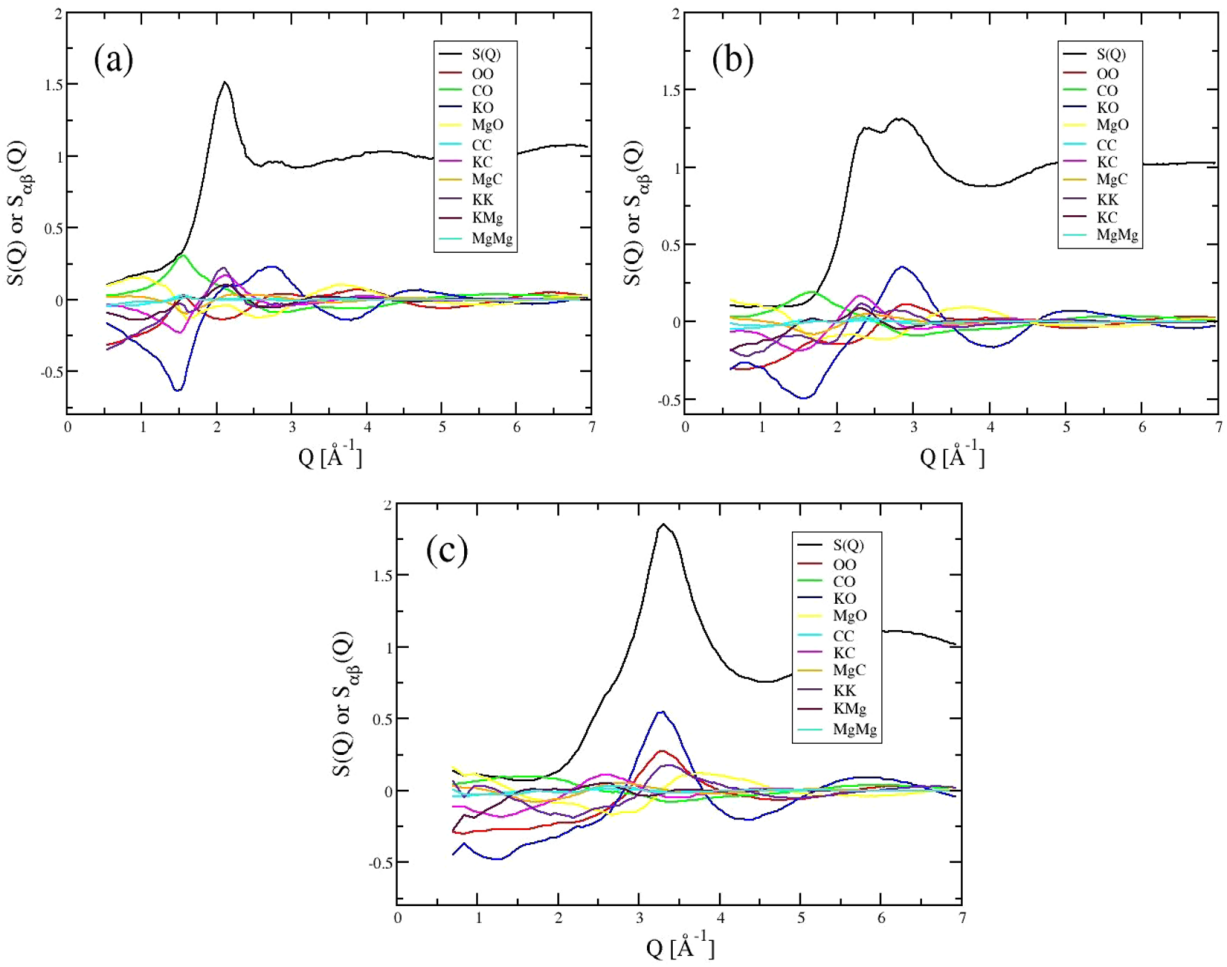
X-ray total structure factors shown with the X-ray weighted contributions from the ten partial structure factors as indicated at (**a**) low, (**b**) medium and (**c**) high densities.

**Figure 3:**
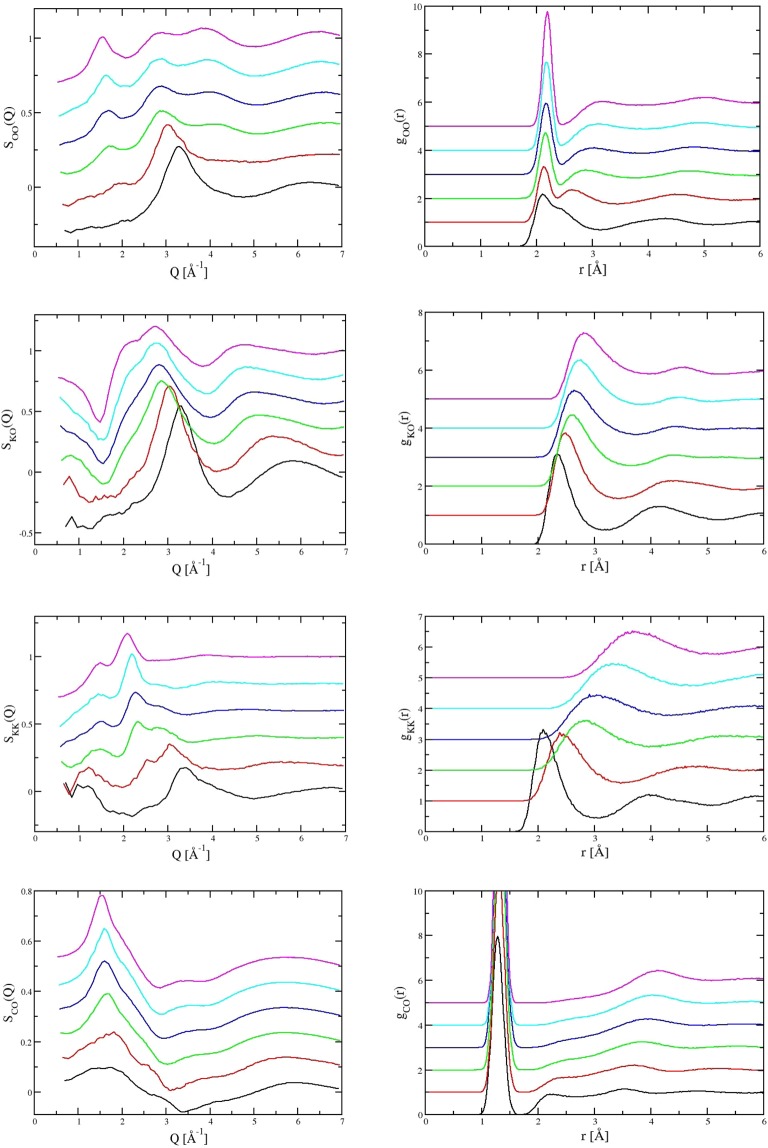
The evolution of the partial structure factors (left panels) and partial radial distribution functions (right panels) for the atom pairs (from top to bottom) O-O, K-O, K-K and C-O obtained from molecular dynamics computer simulation. Each panel shows results at six densities from low density (highest curves) to high density (lowest curves). Successive curves are offset along the abscissa for clarity.

In Fig. 1b *S*(*Q*) functions obtained from simulation are shown for six different densities which can be directly compared to those obtained from X-ray diffraction. These results clearly reproduce the changes seen in the experimental functions and allow us to identify the detailed underlying changes in the partial structural contributions. Figure 2 shows *S*(*Q*) for three pressures, together with the X-ray weighted contributions from each partial structure factor. As predicted, *S*(*Q*) is dominated by the O-O, K-O and K-K pair contributions. The un-weighted trends in these three partial contributions are shown in Fig. 3, together with the change in the the C-O contribution. These partial contributions are shown in both reciprocal and real space. The pressure-dependence of all 10 partial structure factors is shown in the SI (Fig. S4).

The flexibility of the O-O and C-O bonds are clearly important in determining the response of the carbonate liquid structure to high pressure. This is seen most obviously in the changes in the partial O-O contribution in Fig. 2, with the disappearance of the peak at *Q* ~1.5 Å^−1^ and the gradual increase in intensity of a peak at *Q* ~3.0 Å^−1^ with a progressive shift to higher *Q* with pressure. There are changes at higher *Q* in this partial contributions that suggest changes to a more densely packed oxygen sub-structure. The K-O partial contribution shows similar changes with a decrease in the intensity of a shoulder at *Q* ~2.0 Å^−1^ and increase in the intensity of a peak at *Q* ~3 Å^−1^. This overlaps with the O-O peak and dominates the high pressure diffraction pattern. There are shifts in the K-K partial contribution similar to the changes in O-O at high pressure whilst the C-O contribution becomes broader, indicating increased distortion of the local C-O environment.

The un-weighted partial contributions for the O-O, K-O K-K and C-O pairs are shown in both reciprocal and real space in Fig. 3. In real space there are changes in the first O-O peak with the growth of a distinct shoulder at higher *r* that suggests the evolution of two overlapping O-O length-scales. There are also shifts in the oscillations at higher *r*. In the K-O partial function, the nearest-neighbour K-O length-scale shifts to lower *r* whilst there is a similarly significant change in the K-K pair contribution which also shifts to lower *r* with pressure and becomes commensurate with the O-O contribution. The partial radial distribution functions for all ten atom pairs are shown in the SI (Fig. S5)

In the previously suggested ambient pressure glass structure^31^, K^+^ ions act as a “modifier species” while Mg^2+^ cations act as “bridging species” between the carbonate anions. There is relatively little change in the partial contributions from the Mg^2+^ cations in either reciprocal or real space (SI Figs S4 and S5). The Mg-O partial functions remain effectively fixed with pressure, inconsistent with the more traditional concept of a modified polymerized network. Although the main changes in the diffraction data reflect those changes in the partial contributions that dominate the X-ray scattering, the simulations show that these changes are themselves indicative of associated changes in the underlying structure of the carbonate anions. The relationship between the K^+^ cations and the carbonate anions is considerably more complicated and is characterised by structures that result from the strong electrostatic interactions between the oxygen atoms in the carbonates and K^+^ cations, which result in preferential formation of close CO_3_ pairs and the emergence of a second C-O length-scale. This is shown in changes in the C-O partial radial distribution function with pressure. There is the progressive formation of a peak at *r* ~ 2.4 Å as the pressure is increased, evidence of an emergent second length scale, and the formation of a network-structure in the C-O system.

**Figure 4:**
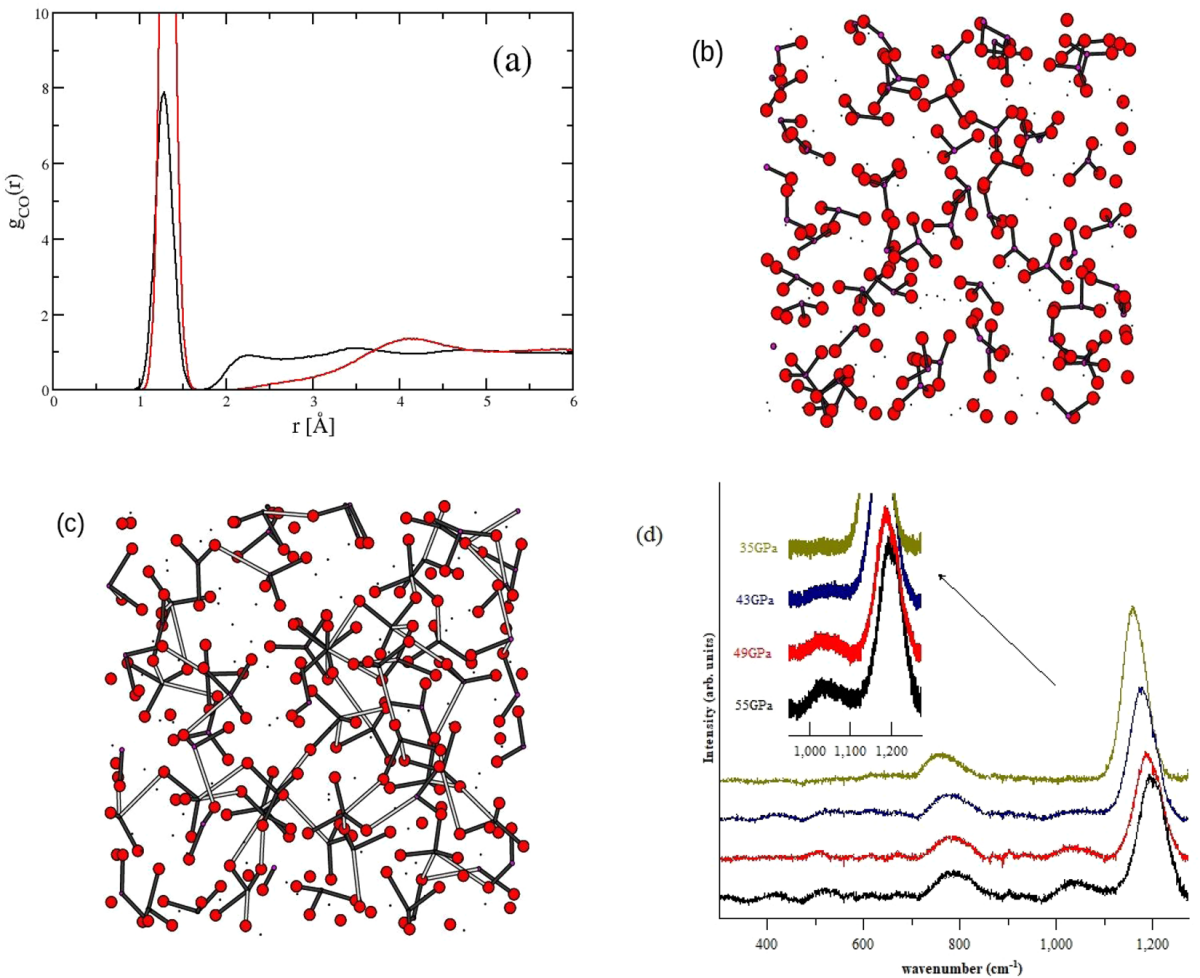
The C-O partial radial distribution function, *g*_*CO*_(*r*), obtained from molecular dynamics computer simulation and shown at high and low pressure (black and red lines resp.) (**a**). Note the emergence of a second length-scale at high pressure at *r* ~ 2.2 Å. Molecular graphics “snapshot” of the carbonate liquid at ambient pressure (**b**) highlighting the C and O atoms only. Black bonds indicate *r*_*CO*_ < 1.7 Å. Molecular graphics “snapshot” at high pressure (**c**) also highlighting the C and O atoms. Black bonds indicate *r*_*CO*_ < 1.7 Å whilst grey bonds highlight 1.7 Å < *r*_*CO*_ < 2.4 Å (*i.e*. the second length-scale). Independent verification of the CO_3+1_ configuration has been obtained from Raman spectroscopy (see SI) which shows the development of a weak peak at ~1040 *cm*^−1^. This peak is discernible at high pressures where the concentration of the CO_3+1_ configurations is expected to be high (**d**).

In Fig. 4a we illustrate the emergence of the second C-O length-scale in the partial pair distribution function at two extremes of density. At ambient pressure the first peak at *r* ~ 1.3 Å is the C-O length-scale in the molecular anion with the C-O length-scale at *r* ~ 4.1 Å representing the distance to the nearest-neighbour anions. At high pressure the second C-O length-scale at *r* ~ 2.4 Å emerges. A molecular dynamics “snapshot” of the ambient pressure configuration (Fig. 4b) shows the molecular anions with bonds drawn at a distance of r _*CO*_ < 1.7 Å. As pressure is applied (Fig. 4a) the first C-O peak shifts to lower *r* with substantive changes at higher *r* with the clear emergence of a second C-O length-scale at *r* ~ 2.2 Å. The molecular graphics “snapshot” of this high pressure configuration is shown in Fig. 4c, highlighting the two length scales. This clearly shows the formation of a network structure in the C-O system which is moderated by the changes in the potassium sub-density and which dominate the first peak in *S*(*Q*) in the high pressure diffraction pattern.

The flexibility of the carbonate anion allows the carbon atom to move out-of-plane and hence allows the coordination shell of carbon to expand and incorporate additional oxygen. This increases the fraction of four-coordinate carbon although the emergence of a second C-O length-scale scale means these four-coordinate (CO_3+1_) configurations will be asymmetric. A similar, second C-O length-scale is reported for (Mg, Fe)CO_3_ carbonate minerals at pressures of 80 GPa^57^. In the K_2_CO_3_-MgCO_3_ glass, the second C-O length-scale is much greater than those reported by Boulard *et al*.^57^ but is also a “three-plus-one” (CO_3+1_) configuration. Neither of these “three-plus-one” anions have the four, symmetric, equidistant bonds of tetrahedral CO_4_ observed for the hard, solid phases of CO_2_ stable above *p* ~25 GPa and the amorphous CO_2_ (“carbonia”) stable above *p* ~40 GPa^16–19^ or the corner sharing CO_4_ rings formed above *p* ~100 GPa in carbonates such as MgCO_3_^58^ or CaCO_3_^59^.

## Discussion

The structure and density of carbonate liquids at ultra-high pressure will influence the physical behaviour of carbonate liquids, the geochemistry of deep Earth processes and the global carbon cycle. For instance, it has been proposed that oceanic crust that has been subducted on the sea-floor can release a carbonate melt as it subducts into the mantle and meets a solidus ledge at 10–21 GPa within the transition zone^9^. It is simply not known whether these liquids will be low enough in density or viscosity to be buoyant and efficiently escape the source or whether their density or high viscosity condemn them to be dragged deeper into the lower mantle by the motion of the subducting slab. Although *in situ* measurements of K_2_CO_3_ and K_2_CO_3_-CaCO_3_ liquid density by Dobson^12^ had initially suggested that carbonate liquids may become very dense at high pressures and may even exceed the density of basaltic liquids, combined sound-speed and density measurements of carbonate liquids^60–62^ show that (K_2_CO_3_) carbonate liquids are highly compressible and although the density of the liquid increases with pressure, this increase is restricted to relatively shallow pressures <4 GPa. At pressures >4 GPa the carbonate liquid densities are lower than those of anorthite and diopside liquids. Extrapolation to higher pressures of the equations of state for K_2_CO_3_^60–63^ and CaCO_3_^64,65^ liquids suggest that the carbonate liquids remain buoyant at transition zone pressure and carbon would not be further subducted into the Lower Mantle for all but the coldest of modern-day slabs. We have derived the equation of state for the carbonate glass studied here using combined ultrasonic interferometry and X-ray radiography (SI Fig. S3)^11^. These experimental density data are used to derive a third-order Birch-Murnaghan equation of state with values of *K*_0_ of 54.53 GPa and ${K^{\prime} }_{0}$ of 4.36. This equation of state indicates that the high pressure (K_2_CO_3_-MgCO_3_) glass has a density similar to that of K_2_CO_3_ liquid when extrapolated to pressures in excess of 20 GPa. Measurements of carbonate liquid viscosity by falling sphere methods of K_2_CO_3_-MgCO_3_^12^, Mg_0.40_Fe_0.09_Ca_0.51_CO_3_^11^ and CaCO_3_ liquids^66^ all indicate that the carbonate liquids are inviscid and whilst there is variation in the viscosity of different carbonate liquids, due to composition and differences in temperature of measurement, the viscosities are 2–3 orders or magnitude lower than basaltic melts and carbonates are therefore expected to have high melt mobility and rapid ascent rates. These measurements suggest a limited influence of pressure on the carbonate liquid viscosity^11,12^. They are, however, restricted to relatively low pressures of ~6 GPa and the present study shows that there are substantial changes in the structure of the high pressure glass with higher pressure (up to 45 GPa) and formation of an extended carbonate network which we correlate with an increase in liquid viscosity which would restrict melt mobility. These structural changes appear continuous and the low-dimensional structures emerge at *p* ~10 GPa which dominate the liquid structure at pressures in excess of 20 GPa. The extent of the low-dimensional carbonate network is very sensitive to temperature and decreases as temperature is increased^13,15^. Furthermore, the formation of the network is correlated with the ionic diffusion coefficients and hence the system viscosity^15^. The diffusion coefficients for potassium, the fastest diffusing species, can be extracted from the simulations and at the two extremes of density *D*_*K*_ decreases from ~10^−4^ to 10^−7^ cm^2^/s at 2500 K. Assuming that the viscosity is proportional to diffusion (Stokes-Einstein) then the viscosity will be expected to increase by three orders of magnitude at this temperature over the pressure range of 0 to 44 GPa. Increased pressure that favours the formation of the emergent networks would also mean that the carbonate liquids become less *fragile* with increasing pressure. Such an increase in the melt viscosity would decrease the ascent rate and would mean that carbonate liquids generated within the transition zone could remain close to their source and not migrate rapidly. There is recent geophysical evidence to support this. Carbonate liquids have a high electrical conductivity and the presence of small amounts of carbonate in the transition zone would contribute to the observed high electrical conductivity in the asthenosphere^6,67^.

The focus of this study thus far has been on the changes in glass structure and the excellent reproduction of the diffraction data by advanced simulation of the equivalent liquids. The results show the emergence of a second length-scale. The diffraction data are, however X-ray weighted and so do not show direct evidence of the CO_3+1_ configuration. Accordingly we have used Raman spectroscopy to demonstrate the presence of CO_3+1_. Raman spectra were collected for a chip of the K_2_CO_3_-MgCO_3_ glass in a diamond anvil cell for pressures of up to 55 GPa. These spectra (SI Fig. S6) clearly show the development of a peak at 1050 cm^−1^ with pressure that is consistent with the presence of CO_3+1_^64,68^. This peak has low intensity and remains stationary as pressure is applied, as would be expected from the weak bonding associated with the emerging, second C-O distance. Furthermore this peak is only apparent at high concentrations of CO_3+1_ at the highest pressures. None the less these Raman data provide an independent verification of the CO_3+1_ configuration, confirming the results of the combined diffraction and simulation study (Fig. 4d).

The composition studied here (K_2_CO_3_-MgCO_3_) differs from the composition of naturally occurring carbonate liquids and their simple analogues. However, there is evidence to suggest that the formation of low-dimensional networks can be extended to other carbonate systems. Studies of molten CaCO_3_ by first principles molecular dynamics (FPMD) simulation show an increased fraction of four-coordinate carbon^65^ consistent with the presence of the different anionic bonding environments proposed by Williams and Knittle^69^. Similarly there is the apparent emergence of a second C-O length-scale in an FPMD study of CaCO_3_ at high pressure (~12 GPa)^64^. A second C-O length-scale also emerges when CO_2_ is dissolved in CaCO_3_^70^. These studies suggest that the formation of low dimensional structures in carbonate liquids (resulting from the flexibility of the carbonate anions) is ubiquitous.

To conclude, we have used advanced high pressure techniques to determine the structure of a rare K_2_CO_3_-MgCO_3_ glass at ultra-high pressures and have used molecular dynamics simulation that uses a flexible carbonate anion to interpret the structural trends. The changes in diffraction patterns largely represent changes in the K-K, K-O and O-O pair correlations. However, there is a complex structural relationship between the carbonate anions and alkali cations, with the most dramatic changes in structure occurring within the carbonate anions themselves. The pressure-induced changes in the flexible carbonate anions, moderated by strong changes in the potassium sub-density, result in the formation of a carbonate network and a change from a molecular to polymerised liquid with an expected increase in carbonate liquid viscosity at high pressures. This gradual change to the CO_3+1_, network structure begins at pressures much lower than CO_4_ formation in CO_2_-V or amorphous CO_2_ or in the solid carbonates MgCO_3_, CaCO_3_ or (Mg, Fe)CO_3_.

The results of this study show that changes in the total structure factor in response to ultra-high pressures are directly related to the emergence of ordering on new length-scales. The excellent description of the high pressure carbonate liquids derives from the relative simplicity of the chemically-motivated structural model, with the development of a carbonate network shown to be responsible for the new length-scales and arising as a direct result of the flexibility of the carbonate anion and the interactions with the potassium cations. The potassium cations become networks-formers at high pressure. The emergence of these networks has significant implications for properties such as viscosity, critical in understanding key geochemical processes.

## Material and Methods

### Glass synthesis

Carbonate glass samples were prepared using a bulk composition of 45:55 (mol%) MgCO_3_ to K_2_CO_3_ which is above a eutectic (~460 °C)^32,33^ in this binary system and easily forms a glass at the quench rate of these experiments (~20 0C/s). Reagent grade (>99.9%) potassium carbonate and natural magnesite (Brumado, Bahia, Brazil; water free by IR) were ground together and loaded into gold tubing (3.8 mm diameter, 10 mm long) and welded shut. The capsule was loaded in a modified rapid quench, Tuttle -type cold seal hydrothermal pressure vessel and then run at 100 MPa. and 780 °C for >6 hrs. Post run, the glass was retrieved from the capsule and stored with desiccant.

### High pressure assembly

To achieve pressures of up to 44 GPa^28^, a specially developed double stage large volume press was used, which incorporates a second-state pair of 1.2 mm culet diamond anvils to achieve high pressures whilst still having a sufficiently large opening to allow X-ray diffraction measurement^28^. The *in situ* energy dispersive X-ray diffraction data were collected on pristine fragments of the carbonate glass that were loaded into a composite gasket, which comprises a cubic boron nitride + epoxy (10:1 in weight ratio) insert in a pre-stressed aluminium alloy gasket a two stage toroidal cell^28^. The sample size was 0.5 mm diameter and 0.2 mm thick. A small piece of gold foil was also included in the gasket for pressure calibration using the equation of state for gold^71^. No pressure medium was used in order to avoid large contributions to the X-ray scattering signal and to prevent interaction between the pressure-medium and the carbonate glass. The second stage assembly was loaded into the first-stage assembly formed from PEEK and MgO sleeves contained in a boron-epoxy gasket. The entire assembly was loaded into a 12 mm diameter, flat-bottomed cup in the first stage anvil of a Paris-Edinburgh press. Pressure was increased by hydraulic ram and with pressures between 6.9 and 96.5 MPa (1000 and 14000 psi).

### EDXRD

The total X-ray structure factor was obtained using the multi-angle energy dispersive technique. This uses a focused, white X-ray beam with 7 × 7 *μ*m size, scattering data is collected on a Ge solid state detector (Canberra) at 2 theta angles of 3.14°, 4.14°, 5.14°, 7.14°. 9.14°, 12.15°, 16.15°, 22.15°, 28.14° and 31.32°, this detector was calibrated using gold peaks at ambient pressure conditions. The total exposure for each pressure point was obtained by normalizing each detector pattern to the white X-ray beam^47^ with further corrections using the optimisation techniques described by Shen *et al*. and Kono *et al*.^11,47^ The energy dispersive patterns for each detector were rescaled and merged to form a Faber-Ziman type total structure factor. In this study we have eliminated the data from the 3.14° detector bank since this clearly showed crystalline peaks from the sample assembly. The scattering intensity in the 31.32° detector bank was very low and these latter data are also eliminated from the subsequent normalization. The individual segments were smoothed by an error weighted spline and scaled to the energy of the primary X-ray beam in the highest angle segment (in this case 28°) (Figs S1 and S2).

### Density

The equation of state for the K-Mg carbonate glass was determined through a series of high pressure ultrasonic measurements, combined with X-ray radiography and EDXRD measurements also performed at sector 16 (APS)^11^. These have been used to determine the density of the carbonate glass with pressure and the equation of state to pressures of up to 10 GPa. A third order Birch-Murnaghan equation of state is fitted using values of *K*_0_ of 54.53 GPa and ${K^{\prime} }_{0}$ of 4.36. The density of the K_2_CO_3_-MgCO_3_ glass is compared with that of liquid K_2_CO_3_ from Liu *et al*.^61,62^ and O’Leary *et al*.^60^. These are also fitted with a third order Birch-Murnaghan equation of state. The *in situ* data from Dobson^12^ are also shown (SI Fig. S3). As discussed by Liu *et al*. the values from Dobson are not consistent with the fusion curves for K_2_CO_3_, which are an independent determination of liquid density. By using a combination of sound speed data and room pressure density measurements the high pressure compressibility data for K_2_CO_3_ is obtained. The best estimate for the value of ${K^{\prime} }_{0}$ is 13.7. With this value the compressibility of K_2_CO_3_ decreases rapidly with pressure and becomes comparable with liquid anorthite and diopside. The densities for liquid CaCO_3_ derived from First Principles Molecular Dynamics (FPMD) simulations^65^ are also shown together with the density of aragonite (Fig. S3).

### Molecular dynamics simulation

Molecular dynamics simulations have been performed on carbonate liquids using a potential developed by Tissen and Janssen of the Born-Huggins-Mayer form^49^. The systems studied contain 1536 oxygen, 512 carbon, 512 K^+^ and 256 Mg^2+^ atoms/ions. The trigonal geometry of the carbonate anion is imposed by employing harmonic springs that act between C-O and O-O pairs^13^ with the respective spring force constants taken from previous work focussed on sulphates^72^. In the simulations carried out here we employ a fixed charge distribution on the anions, with *q*_*O*_ = −1.18*e* and *q*_*C*_ = +1.54*e*. The cations carry their formal valence charges. The simulations have been carried out at a fixed temperature (of *T* = 1800 K) and at constant volume, with the simulations performed at different densities to evaluate the influence of pressure on the liquid carbonate structure. At each density simulations of the order of 500 ps are performed. The number densities used are (in molecules/Å^3^); 0.0122, 0.00986, 0.00794, 0.00724, 0.00657, and 0.00572.

*F*^*x*^(*Q*) was generated by combining the partial(Ashcroft-Langreth) structure factors (of which there are ten for the four component system). These were calculated directly from the Fourier components of the ion densities, *S*_*αβ*_(*Q*) = 〈*A*_α_^*^(*Q*)*A*_*β*_(*Q*)〉, where ${A}_{\alpha }(Q)=\frac{1}{\sqrt{{N}_{\alpha }}}\mathop{\sum }\limits_{j=1}^{{N}_{\alpha }}\,\exp (i{\bf{Q}}.{{\bf{r}}}^{j})$ (Figs S4 and S5). Total X-ray structure factors were constructed from weighted sums of these partial structure factors using X-ray form factors taken from standard sources^73^.

1 $${F}^{x}(Q)=\sum _{\alpha }\sum _{\beta }{f}_{\alpha }(k){f}_{\beta }(k)\sqrt{{c}_{\alpha }{c}_{\beta }}({S}_{\alpha \beta }(k)-{\delta }_{\alpha \beta }).$$

### Raman spectroscopy

High-pressure micro-Raman spectroscopy measurements were made for the glass in a Princeton-type symmetric diamond anvil cell (DAC) using low fluorescence type-II diamonds with 300 *μ*m culet diameters. The glass was crushed immediately after preparation and a chip of this material was loaded into a 100 *μ*m hole laser-drilled into a Re gasket pre-indented to a thickness of ~50 *μ*m. For pressure calibration, a 5 *μ*m diameter ruby standard was loaded at the edge of the sample chamber. *In situ* Raman spectra were acquired using a Jobin-Yvon T64000 triple spectrometer operating in confocal mode. A 532 nm laser was focused to a 3–5 *μ*m spot on the sample and Raman spectra taken from 200 to 1300 cm^−1^. Pressure was determined both from the ruby scale^74^ and from the shift of the diamond singlet peak at the culet surface^75^ (Fig. S6).

## Supplementary information

Supplementary information

## References

[1] Jones, A. P., Genge, M. &Carmody, L. Carbonate Melts and Carbonatites. In Hazen, R. M., Jones, A. P. & Baross, J. A. (ed.) Carbon in Earth, vol. 75 of *Reviews in Mineralogy & Geochemistry*, 289–322 (2013).

[2] Woolley AR, Church AA (2005). Extrusive carbonatites: A brief review. Lithos.

[3] Mitchell RH (2005). Carbonatites and carbonatites and carbonatites. Canadian Mineralogist.

[4] Bailey D (1993). Carbonate Magmas. Journal of the Geological Society.

[5] Tuttle, O. F. & Gittins, J. *Carbonatites* (Interscience publishers, New York, 1966).

[6] Gaillard F, Malki M, Iacono-Marziano G, Pichavant M, Scaillet B (2008). Carbonatite Melts and Electrical Conductivity in the Asthenosphere. Science.

[7] Sifre D (2014). Electrical conductivity during incipient melting in the oceanic low-velocity zone. Nature.

[8] Sifre D, Hashim L, Gaillard F (2015). Effects of temperature, pressure and chemical compositions on the electrical conductivity of carbonated melts and its relationship with viscosity. Chemical Geology.

[9] Thomson AR, Walter MJ, Kohn SC, Brooker RA (2016). Slab melting as a barrier to deep carbon subduction. Nature.

[10] Hudspeth J, Sanloup C, Kono Y (2018). Properties of molten CaCO_3_ at high pressure. Geochemical Perspective Letters.

[11] Kono, Y. *et al*. Ultralow viscosity of carbonate melts at high pressures. *Nature Communications* **5** (2014). 10.1038/ncomms6091 25311627

[12] Dobson D (1996). *In-situ* measurement of viscosity and density of carbonate melts at high pressure. Earth and Planetary Science Letters.

[13] Wilding, M. C. *et al*. Low-Dimensional Network Formation in Molten Sodium Carbonate. *Scientific Reports* **6** (2016). 10.1038/srep24415 PMC4832186 27080401

[14] Wilding MC (2017). The structure of liquid alkali nitrates and nitrites. Physical Chemistry Chemical Physics.

[15] Wilson M (2018). Structure and Liquid Fragility in Sodium Carbonate. Journal of Physical Chemistry A.

[16] Santoro M (2006). Amorphous silica-like carbon dioxide. Nature.

[17] Santoro M, Gorelli FA (2006). High pressure solid state chemistry of carbon dioxide. Chemical Society Reviews.

[18] Santoro M (2011). Silicon carbonate phase formed from carbon dioxide and silica under pressure. Proceedings of the National Academy of Sciences of the United States of America.

[19] Santoro M., Gorelli F. A., Bini R., Haines J., Cambon O., Levelut C., Montoya J. A., Scandolo S. (2012). Partially collapsed cristobalite structure in the non molecular phase V in CO2. Proceedings of the National Academy of Sciences.

[20] Gregoryanz, E., Goncharov, A., Hemley, R. & Mao, H. High-pressure amorphous nitrogen. *Physical Review B* **64** (2001).

[21] Rosa AD (2016). *In situ* characterization of liquid network structures at high pressure and temperature using X-ray absorption spectroscopy coupled with the Paris-Edinburgh press. High Pressure Research.

[22] Sanloup C (2013). Structure and density of molten fayalite at high pressure. Geochimica et Cosmochimica Acta.

[23] Sanloup C (2013). Structural change in molten basalt at deep mantle conditions. Nature.

[24] Drewitt James W E, Jahn Sandro, Sanloup Chrystèle, de Grouchy Charlotte, Garbarino Gaston, Hennet Louis (2015). Development of chemical and topological structure in aluminosilicate liquids and glasses at high pressure. Journal of Physics: Condensed Matter.

[25] Zeidler, A. *et al*. Density-driven structural transformations in B_2_O_3_ glass. *Physical Review B* **90** (2014).

[26] Zeidler, A. *et al*. High-Pressure Transformation of SiO_2_ Glass from a Tetrahedral to an Octahedral Network: A Joint Approach Using Neutron Diffraction and Molecular Dynamics. *Physical Review Letters* **113** (2014). 10.1103/PhysRevLett.113.135501 25302900

[27] Salmon Philip S, Drewitt James W E, Whittaker Dean A J, Zeidler Anita, Wezka Kamil, Bull Craig L, Tucker Matthew G, Wilding Martin C, Guthrie Malcolm, Marrocchelli Dario (2012). Density-driven structural transformations in network forming glasses: a high-pressure neutron diffraction study of GeO2glass up to 17.5 GPa. Journal of Physics: Condensed Matter.

[28] Kono Y (2016). Ultrahigh-pressure polyamorphism in GeO_2_ glass with coordination number >6. Proceedings of the National Academy of Sciences of the United States of America.

[29] Eitel W, Skaliks W (1929). Double carbonates of alkalis and alkaline earths. Zeitschrift fr Anorganische und Allgemeine Chemie.

[30] Sharma SK, Simons B (1980). Raman study of k_2_co_3_-mgco_3_ glasses. Carnegie Institute of Washington Yearbook.

[31] Genge M, Price G, Jones A (1995). Molecular Dynamics Simulations of CaCO_3_ Melts to Mantle Pressures and Temperatures - Implications for Carbonatite Magmas. Earth and Planetary Science Letters.

[32] Ragone SE, Datta RK, Roy DM, Tuttle OF (1966). The system potassium carbonate-magnesium carbonate. The Journal of Physical Chemistry.

[33] Datta RK, Roy DM, Faile SP, Tuttle OF (1964). Glass formation in carbonate systems. Journal of The American Ceramic Society.

[34] Forland T, Weyl WA (1950). Formation of a sulfate glass. Journal of the American Ceramic Society.

[35] MacFarlane, D. R. Attempted glass formation in pure khso_4_. *Communications of the American Ceramic Society* C–28 (1984).

[36] van Uitert LG, Grodkiewicz WH (1971). Nitrate glasses. Materials Research Bulletin.

[37] Genge M, Jones A, Price G (1995). An Infrared and Raman Study of Carbonate Glasses - Implications for the Structure of Carbonatite Magmas. Geochimica et Cosmochimica Acta.

[38] Fine G, Stopler E (1985). The Speciation of Carbon Dioxide in Sodium Aluminosilicate Glasses. Contributions to Mineralogy and Petrology.

[39] Kohn R, Brooker SC, Dupree R (1991). C-13 MAS NMR - A Method for Studying CO_2_ Speciation in Glasses. Geochimica et Cosmochimica Acta.

[40] Kubicki J, Stolper E (1995). Structural roles of CO_2_ and CO_3_(2−) in fully polymerized Sodium Aluminosilicate melts and glasses. Geochimica et Cosmochimica Acta.

[41] Brooker R, Kohn S, Holloway J, McMillan P, Carroll M (1999). Solubility, speciation and dissolution mechanisms for CO_2_ in melts on the NaAlO_2_-SiO_2_ join. Geochimica et Cosmochimica Acta.

[42] Brooker, R., Kohn, S., Holloway, J. & McMillan, P. Structural controls on the solubility of CO_2_ in silicate melts Part I: bulk solubility data. *Chemical Geology* **174**, 225–239 6th International Silicate Melt Workshop, FRANCE, APR 13-17, 1999 (2001).

[43] Brooker, R., Kohn, S., Holloway, J. & McMillan, P. Structural controls on the solubility of CO_2_ in silicate melts Part II: IR characteristics of carbonate groups in silicate glasses. *Chemical Geology* **174**, 241–254 6th International Silicate Melt Workshop, France, Apr 13–17, 1999 (2001).

[44] Kono Yoshio, Kenney-Benson Curtis, Shibazaki Yuki, Park Changyong, Wang Yanbin, Shen Guoyin (2015). X-ray imaging for studying behavior of liquids at high pressures and high temperatures using Paris-Edinburgh press. Review of Scientific Instruments.

[45] Wang Y, Shen G (2014). High-pressure experimental studies on geo-liquids using synchrotron radiation at the Advanced Photon Source. Journal of Earth Science.

[46] Sakamaki T (2014). Contrasting sound velocity and intermediate-range structural order between polymerized and depolymerized silicate glasses under pressure. Earth and Planetary Science Letters.

[47] Shen G, Prakapenka V, Rivers M, Sutton S (2003). Structural investigation of amorphous materials at high pressures using the diamond anvil cell. Review of Scientific Instruments.

[48] Cartwright JHE (2012). Calcium Carbonate Polyamorphism and Its Role in Biomineralization: How Many Amorphous Calcium Carbonates Are There?. Angewandte Reviews.

[49] Tissen JTWM, Janssen. GJM (1990). Molecular-dynamics simulation of molten alkali carbonates. Molecular Physics..

[50] Ribeiro M (2000). First sharp diffraction peak in the fragile liquid ca0.4k0.6(no3)1.4. Physical Review B.

[51] Ribeiro M, Almeida L (2000). Validating a polarizable model for the glass-forming liquid ca0.4k0.6(no3)1.4 by ab initio calculations. Journal of Chemical Physics.

[52] Ribeiro M (2001). Ionic dynamics in the glass-forming liquid ca0.4k0.6(no_3_)1.4: A molecular dynamics study with a polarizable model. Physical Review B.

[53] Rappe A, Goddard W (1991). Charge equilibration for molecular dynamics simulations. Journal of Physical Chemistry.

[54] Liu Y-P, Kim K, Berne B, Friesner R, Rick S (1998). Constructing ab initio force fields for molecular dynamics simulations. Journal of Chemical Physics.

[55] Rick S, Stuart S, Berne B (1994). Dynamical fluctuating charge force fields: Application to liquid water. Journal of Chemical Physics.

[56] Costa M (2008). Molecular dynamics of molten li_2_co_3_-k_2_co_3_. Journal of Molecular Liquids.

[57] Boulard, E., Pan, D., Galli, G., Liu, Z. &Mao, W. L. Tetrahedrally coordinated carbonates in Earth’s lower mantle. *Nature Communications* **6** (2015). 10.1038/ncomms7311 25692448

[58] Oganov AR, Ono S, Ma Y, Glass CW, Garcia A (2008). Novel high-pressure structures of MgCO_3_, CaCO_3_ and CO_2_ and their role in Earth’s lower mantle. Earth and Planetary Science Letters.

[59] Ono S, Kikegawa T, Ohishi Y (2007). High-pressure transition of CaCO_3_. American Mineralogist.

[60] O’Leary, M. C., Lange, R. A. &Ai, Y. The compressibility of CaCO_3_-Li_2_CO_3_-Na_2_CO_3_-K_2_CO_3_ liquids: application to natrocarbonatite and CO_2_-bearing nephelinite liquids from Oldoinyo Lengai. *Contributions to Mineralogy and Petrology* **170** (2015).

[61] Liu Q, Tenner TJ, Lange RA (2007). Do carbonate liquids become denser than silicate liquids at pressure? Constraints from the fusion curve of K_2_CO_3_ to 3.2 GPa. Contributions to Mineralogy and Petrology.

[62] Liu Q, Lange R (2003). New density measurements on carbonate liquids and the partial molar volume of the CaCO_3_ component. Contributions to Mineralogy and Petrology.

[63] Li Z, Li J, Lange R, Liu J, Mintzer B (2017). Determination of calcium carbonate and sodium carbonate melting curves up to Earth’s transition zone pressures with implications for the deep carbon cycle. Earth and Planetary Science Letters.

[64] Vuilleumier R, Seitsonen A, Sator N, Guillot B (2014). Structure, equation of state and transport properties of molten calcium carbonate (CaCO_3_) by atomistic simulations. Geochimica et Cosmochimica Acta.

[65] Zhang Z, Liu Z (2015). High pressure equation of state for molten CaCO_3_ from first principles simulations. Acta Geochimica.

[66] Stagno, V., Stropponi, V., Kono, Y., Manning, C. E. &Irifune, T. Experimental determination of the viscosity of Na_2_CO_3_ melt between 1.7 and 4.6 GPa at 1200–1700 °C; implication for the rheology of carbonatite magmas in the Earth’s upper mantle. *Chemical Geology* **501** (2018).

[67] Caricchi L, Gaillard F, Mecklenburgh J, Trong EL (2011). Experimental determination of electrical conductivity during deformation of melt-bearing olivine aggregates: Implications for electrical anisotropy in the oceanic low velocity zone. Earth and Planetary Science Letters.

[68] Vennari CE, Williams Q (2018). A novel carbon bonding environment in deep mantle high pressure dolomite. American Mineralogist.

[69] Williams Q, Knittle E (2003). Structural complexity in carbonatite liquid at high pressures. Geophysical Research Letters.

[70] Corradini D, Coudert F-X, Vuilleumier R (2016). Carbon dioxide transport in molten calcium carbonate occurs through an oxo-Grotthuss mechanism via a pyrocarbonate anion. Nature Chemistry.

[71] Tsuchiya T (2003). First-principles prediction of the p-v-t equation of state of gold and the 660-km discontinuity in earth’s mantle. Journal of Geophysical Research.

[72] Parfitt DC (2005). High-pressure forms of lithium sulphate: Structural determination and computer simulation. Physical Review B.

[73] Cromer, D. T. & Waber, J. T. *International tables for X-ray Crystallography* (Kynoch Press, Birmingham, 1974).

[74] Mao H, Xu J, Bell P (1986). Calibration of the ruby pressure gauge to 800-kbar under quasi-hydrostatic conditions. Journal of Geophysical Research-Solid Earth and Planets.

[75] Walter MJ (2015). The stability of hydrous silicates in Earth’s lower mantle: Experimental constraints from the systems MgO-SiO_2_-H_2_O and MgO-Al_2_O_3_-SiO_2_-H_2_O. Chemical Geology.

