# Supplementary material for: CO3+1 network formation in ultra-high pressure carbonate liquids
Source: Sci Rep. 2019 Oct 28;9:15416. doi: 10.1038/s41598-019-51306-6 (PMC6817860; doi:10.1038/s41598-019-51306-6)
Supplement: Supplementary file 1 — Supplementary information [file 41598_2019_51306_MOESM1_ESM.pdf]

CO_{3+1} network formation in ultra-high pressure carbonate liquids

Martin Wilding[a],[1,†] Mark Wilson[c],[2] Yoshio Kono[d],[3,¶] James W. E. Drewitt[f],[4] Richard A. Brooker[g],[4] Paul A. Bingham[h],[1] and John B. Parise[i5]

[1]*Materials and Engineering Research Institute,*
Sheffield Hallam University, Howard Street,Sheffield S1 1WB, UK

[2]*Department of Chemistry, Physical and Theoretical Chemistry Laboratory,*
University of Oxford, South Parks Road, Oxford OX1 3QZ, UK

[3]*HPCAT, Geophysical Laboratory, Carnegie Institute of Washington,*
9700 S. Cass Avenue, Argonne, IL 60439, USA

[4]*School of Earth Sciences, University of Bristol,*
Queens Road, Wills Memorial Building, Bristol BS8 1RJ, UK

[5]*SUNY, Stony Brook, NY 11794, USA*

---

[a] martin.wilding@manchester.ac.uk
[c] mark.wilson@chem.ox.ac.uk
[d] kono.yoshio.rj@ehime-u.ac.jp
[f] james.drewitt@bristol.ac.uk
[g] richard.brooker@bristol.ac.uk
[h] p.a.bingham@shu.ac.uk
[i] john.parise@stonybrook.edu

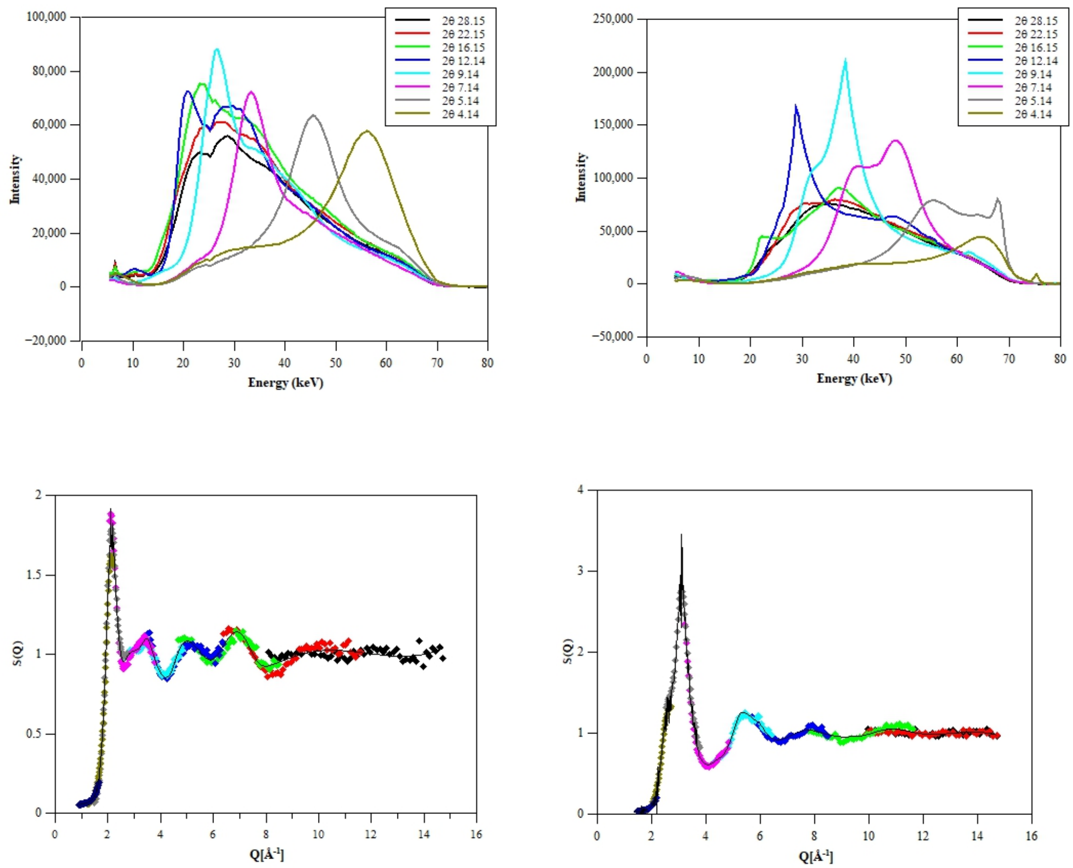

FIG. 1. Scattering intensity as a function of energy for each detector segment from sector 16BM for glass samples at the two extremes of pressure (ambient pressure - upper panels, high pressure (44GPa) - lower panels). The contribution from each detector is then combined and used to generate an error-weighted spline fit presented as the total structure factor, $S(Q)$. The $S(Q)$ data for both pressures are shown together with the spline-smoothed $S(Q)$ in the left panels with the intensity data shown in the right panels.

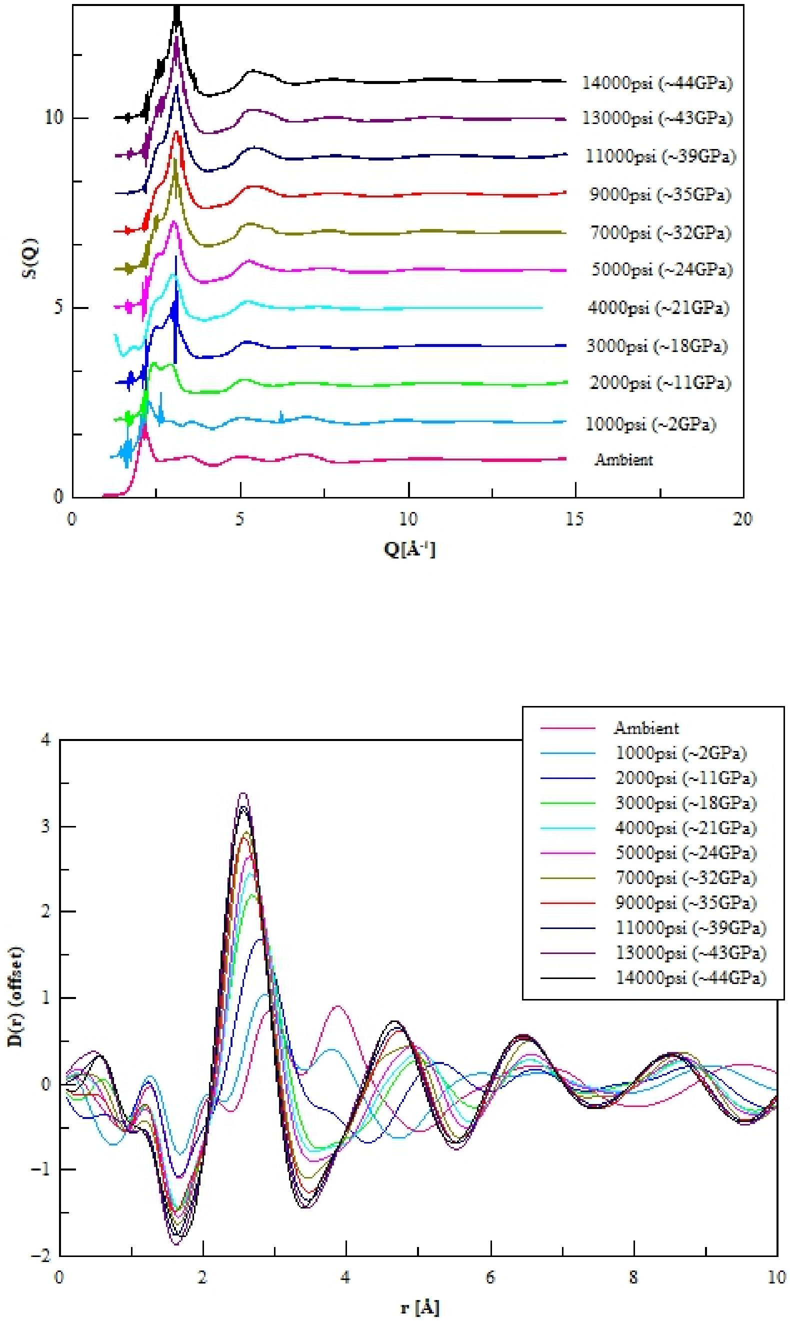

FIG. 2. Spline smoothed $S(Q)$ data (left panel) for the K_2CO_3-$MgCO_3$ glass for pressures up to 44GPa. Although there are "spikes" in the data as a function of the spline smoothing at the detector overlaps these do no correlate with changes in the underlying structure. The right panel shows the Fourier transform to real space of the S(Q) data presented as $D(r) = r(G(r) - 1)$, the total differential distribution function.

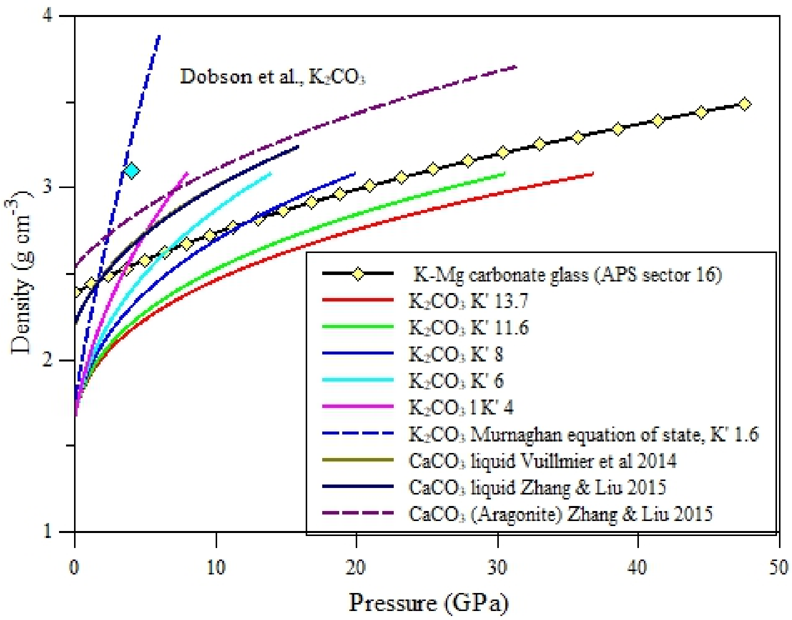

FIG. 3. Density data for the K_2CO_3-$MgCO_3$ glass (Wilding, unpublished), obtained from ultrasonic measurements combined with X-ray radiography and also performed at 16BMB. These data fitted with a third order Birch-Murnaghan equation of state (K_0 = 53.54GPa, K'=4.36). This is compared with density of K_2CO_3liquid also derived from a third order Birch-Murnaghan equation of state with various values of K' and a Murnaghan equation of state for $K' = 1.6$ (see Liu *et al*? ? for details). Values of the density of K_2CO_3 from Dobson? are shown together with data for $CaCO_3$ liquids? and aragonite? .

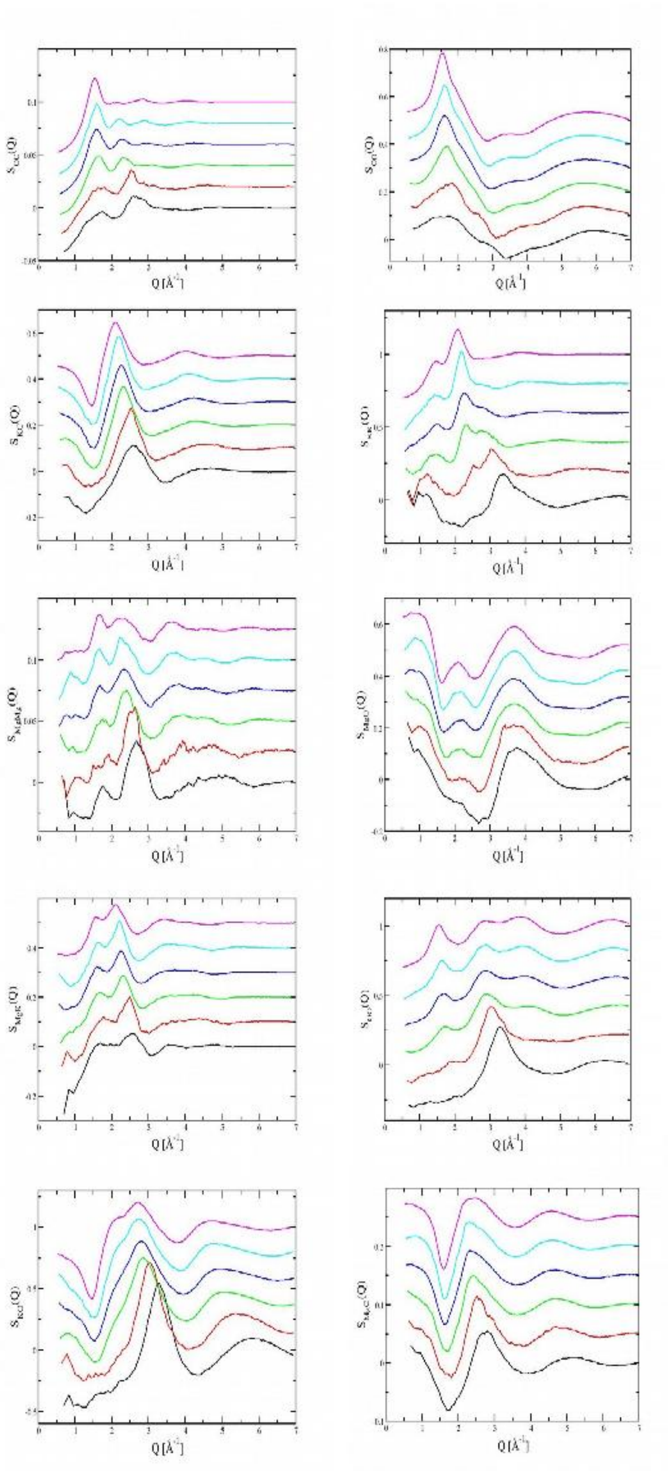

FIG. 4. The evolution of the ten partial structure factors for the atom pairs indicated on the abscissa, obtained from molecular dynamics computer simulation. Each panel shows results at six densities from low density (highest curves) to high density (lowest curves). Successive curves are [illegible]

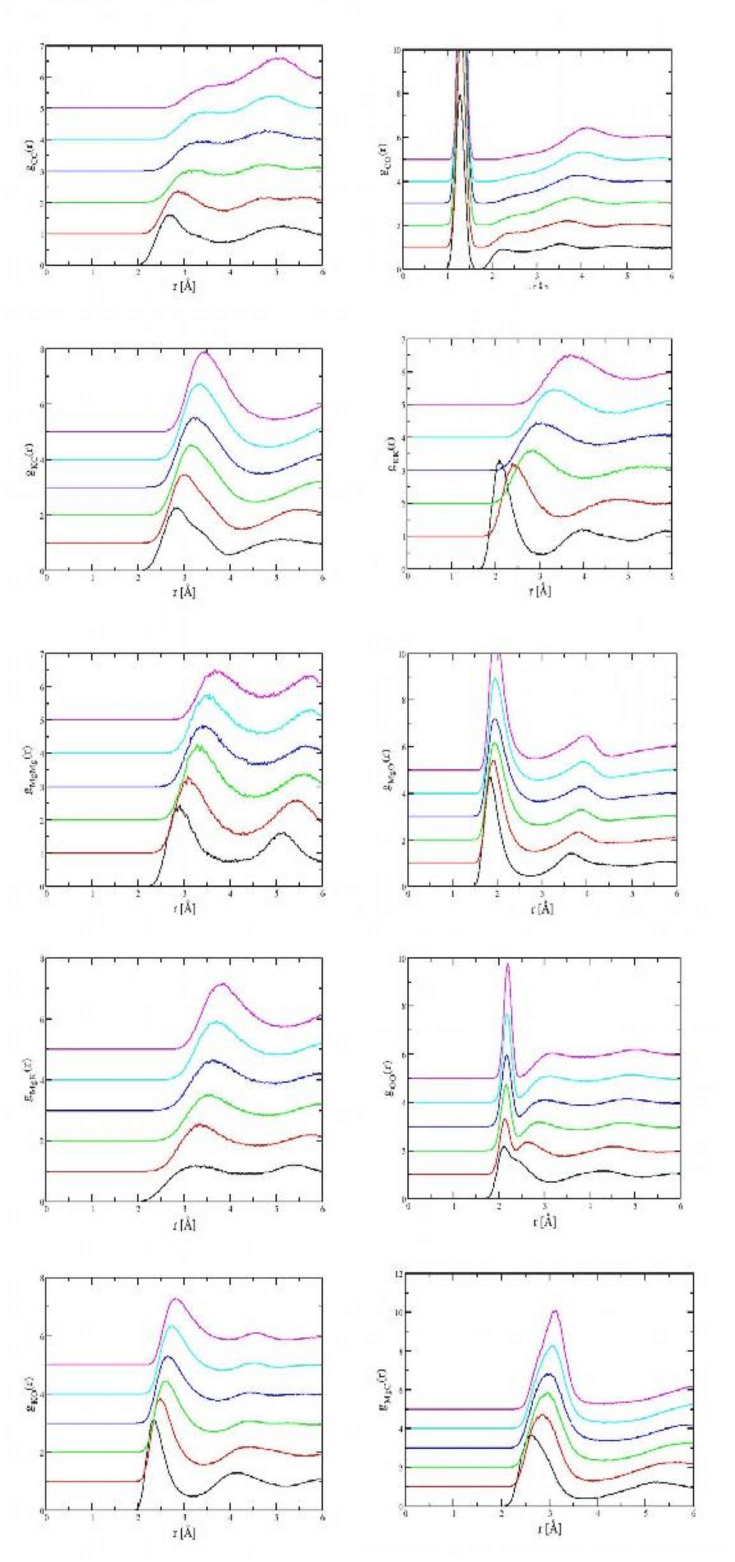

FIG. 5. The evolution of the ten partial radial distribution functions for the atom pairs indicated on the abscissa, obtained from molecular dynamics computer simulation. Each panel shows results at six densities from low density (highest curves) to high density (lowest curves). Successive curves

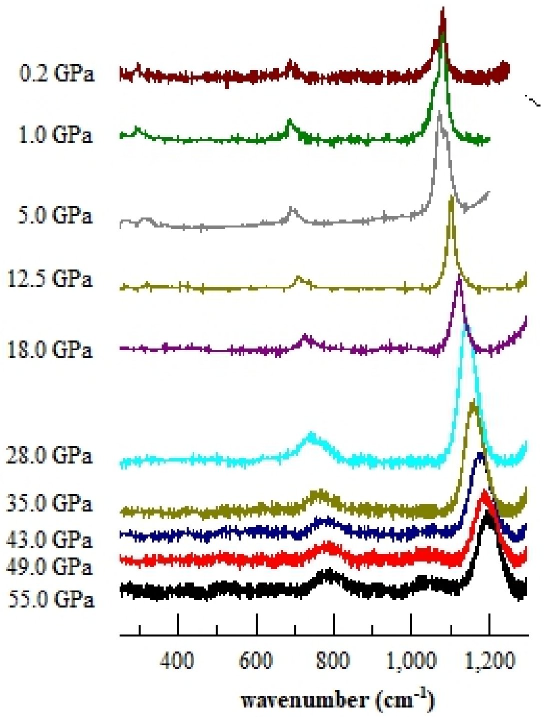

FIG. 6. Raman spectra collected *in situ* for K_2CO_3-$MgCO_3$ glass measured in a diamond anvil cell for pressures up to 55GPa. The left panel shows the changes in Raman spectra as pressure is increased form 0.2 to 55GPa. The main in-plane carbonate bending mode at $\sim$ 1100 cm^{-1} is split and suggests two carbonate populations at ambient pressure, this peak becomes broader and shifts to higher frequency with pressure. At high pressure, a weak peak at $\sim$ 1040 cm^{-1} emerges. This peak reflects the presence of the CO_{3+1} that results from the development of the second C-O length scale and is consistent with the a CO_{3+1} configuration seen in high pressure Raman spectroscopy studies of dolomite[?] . These Raman spectra provide an independent verification of the presence of the CO_{3+1} configuration in the K_2CO_3-$MgCO_3$ glass, consistent with the combined diffraction and simulation.
